# Genome-wide data (ChIP-seq) enabled identification of cell wall-related and aquaporin genes as targets of tomato ASR1, a drought stress-responsive transcription factor

**DOI:** 10.1186/1471-2229-14-29

**Published:** 2014-01-14

**Authors:** Martiniano M Ricardi, Rodrigo M González, Silin Zhong, Pía G Domínguez, Tomas Duffy, Pablo G Turjanski, Juan D Salgado Salter, Karina Alleva, Fernando Carrari, James J Giovannoni, José M Estévez, Norberto D Iusem

**Affiliations:** 1 Instituto de Fisiología, Biología Molecular y Neurociencias (IFIByNE)-CONICET, Buenos Aires, Argentina; 2 Boyce Thompson Institute for Plant Research, Tower Road, Cornell University, Ithaca, NY, USA; 3 Instituto de Biotecnología – INTA, Hurlingham, Provincia de Buenos Aires, Argentina; 4 Departamento de Computación, Facultad de Ciencias Exactas y Naturales, Universidad de Buenos Aires, Buenos Aires, Argentina; 5 Instituto de Biodiversidad y Biología Experimental (IBBEA, CONICET-UBA), Buenos Aires, Argentina; 6 Department of Plant Biology, Cornell University, Ithaca, NY, USA; 7 Departamento de Fisiología, Biología Molecular y Celular, Facultad de Ciencias Exactas y Naturales, Universidad de Buenos Aires, Buenos Aires, Argentina

**Keywords:** Tomato, ASR1, ChIP-seq, Water stress, Cell wall, Aquaporin

## Abstract

**Background:**

Identifying the target genes of transcription factors is important for unraveling regulatory networks in all types of organisms. Our interest was precisely to uncover the spectrum of loci regulated by a widespread plant transcription factor involved in physiological adaptation to drought, a type of stress that plants have encountered since the colonization of land habitats 400 MYA. The regulator under study, named ASR1, is exclusive to the plant kingdom (albeit absent in *Arabidopsis*) and known to alleviate the stress caused by restricted water availability. As its target genes are still unknown despite the original cloning of Asr1 cDNA 20 years ago, we examined the tomato genome for specific loci interacting *in vivo* with this conspicuous protein.

**Results:**

We performed ChIP followed by high throughput DNA sequencing (ChIP-seq) on leaves from stressed tomato plants, using a high-quality anti-ASR1 antibody. In this way, we unraveled a novel repertoire of target genes, some of which are clearly involved in the response to drought stress. Many of the ASR1-enriched genomic loci we found encode enzymes involved in cell wall synthesis and remodeling as well as channels implicated in water and solute flux, such as aquaporins. In addition, we were able to determine a robust consensus ASR1-binding DNA motif.

**Conclusions:**

The finding of cell wall synthesis and aquaporin genes as targets of ASR1 is consistent with their suggested role in the physiological adaptation of plants to water loss. The results gain insight into the environmental stress-sensing pathways leading to plant tolerance of drought.

## Background

Plant species in arid zones are constantly exposed to drought stress [1]. Tolerance to such water deficits most likely occurred in organisms like bryophyte mosses and was evolutionarily important during the conquest of land by plants [2,3]. Proteins of the LEA superfamily are part of the molecular response to this stressful environment [4] and are classified into groups based on amino acid sequence motifs [5]. Despite the various roles suggested for LEA proteins, their precise functions have not been fully revealed.

The widespread (albeit absent in *Arabidopsis*) ASR proteins (Abscisic, Stress, Ripening) are considered to be a subgroup of the LEA superfamily [6,7]. From an evolutionary standpoint, we previously reported the locus Asr2 to have been a target of positive selection in dry habitats, at least in species of the *Solanum* genus [8,9]. Regarding biochemical function, the paralogous Asr1, cloned as long as 20 years ago (GenBank accession number L08255) [10], encodes a 14-kDa polypeptide (ASR1) proposed to act as both a chaperone [11] and a transcription factor (TF) [12]. However, no target genes from tomato have been reported for ASR1 albeit at the beginning and even at completion of this work, two target genes from other species had been identified: i) a sugar transport gene in *Vitis vinifera* (grape) [13] and ii) ABI4 in transgenic *Arabidopsis thaliana*[14]. Given the complexity of the drought stress response in general [15,16], we have long suspected that a great deal of targets exist in the large tomato genome, which contains as many as 34,771 protein-coding genes [17].

Therefore, we were convinced that it was worthwhile pursuing the challenge to identify the direct target genes of ASR1, our regulator of interest, which is thought to control the downstream network necessary for cellular adjustment to water loss. We believed that knowledge on this particular “targetome” would generate valuable mechanistic insights into the genetic program leading to such a physiological adaptation. To achieve this goal, we carried out ChIP-sequencing (ChIP-seq), a strategy that combines chromatin immunoprecipitation (ChIP) with massively parallel (throughput) DNA sequencing to identify the *in vivo* binding sites of DNA-associated proteins, including TFs. As it proved to be useful to map global binding sites precisely for any nuclear protein of interest believed to associate with chromatin, ChIP-seq has emerged as a powerful tool in eukaryotes, particularly in mammals, including humans [18,19], and plants [18-20]. In this way and using a high-quality anti-ASR1 antibody and advanced bioinformatics tools, we generated ChIP-seq data that allowed us to assemble a genome-wide high-resolution DNA-binding map of ASR1, highlighting plant genes that appear to be logically associated with the drought stress response, namely those encoding aquaporins and those associated with the cell wall.

## Results

### The size of the immunoprecipitated fragments (input for ChIP) and quality assessment of the affinity-purified anti-ASR1 antibody

**Figure 1:**
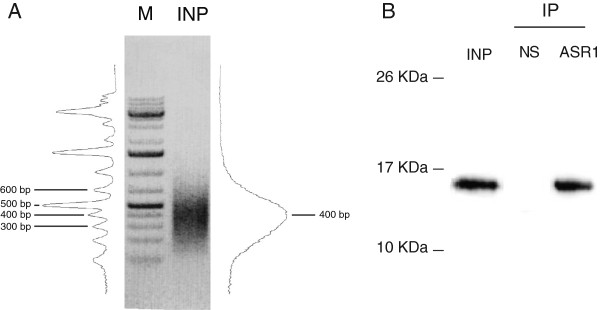
**Library construction and quality assessment of the antibody. A)** Determination of the DNA fragment sizes in input chromatin samples by agarose gel electrophoresis. The gel was stained with ethidium bromide and the band intensity was quantified with ImageJ software. M indicates molecular weight markers (100-bp ladder). **B)** Western blot of the ASR1-immunoprecipitated DNA samples subjected to the ChIP protocol (without crosslinking) using the pure specific anti-ASR1 antibody (ASR1) or an irrelevant, non-specific antibody (NS) and a secondary antibody that recognizes only native immunoglobulins to prevent the undesired detection of immunoglobulins previously used for immunoprecipitation (which denatured on the gel during electrophoresis). INP indicates the input chromatin sample.

After shearing DNA through sonication of lysed nuclei, we determined the average size of the resulting DNA fragments by means of gel electrophoresis. They were approximately 400 bp (Figure 1A), a suitable size for input DNA for subsequent ChIP and library construction.

After the ASR1 protein was successful purified (Additional file 1: Figure S1), an anti-ASR1 antibody was raised in rabbits, affinity-purified and checked via a dot blot (Additional file 1: Figure S2). The immunoprecipitation (IP) ability of this polyclonal anti-ASR1 antibody was tested by performing a preliminary IP assay followed by SDS-PAGE and a Western blot. As expected, we were able to detect a clear single band corresponding to ASR1 (14 kDa) both in samples precipitated with the specific antibody alone as well as in whole chromatin (Figure 1B).

Once the quality of the antibody and the size of the sheared DNA fragments were assessed, we performed the ChIP protocol (see Methods section for details).

### Anti-ASR1 ChIP followed by deep sequencing

**Figure 2:**
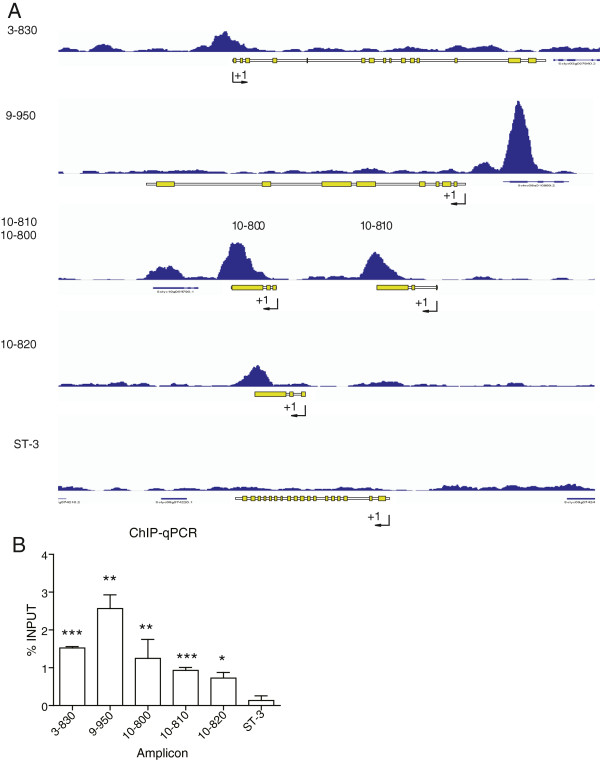
**Visual verification of the peaks and individual validation. A)** A histogram indicating the number of reads of 150-bp window DNA sequences along five selected genomic regions (3–830, 9–950, 10–800, 10–810 and 10–820) where statistically significant peaks were observed. The corresponding gene organization in exons (boxes) and introns is also shown. The +1 arrow indicates the sense of transcription. ST-3 yielded no peaks. **B)** qPCR for the same five selected sequences present in peaks from ChIP-seq. For each locus, the % INPUT is shown. The ST-3 amplicon was used as a negative control. Amplicon names are abbreviations for gene codes (see Table 2, which also shows gene function). Primers are listed in Additional file 1: Table S1. *p < 0.05, **p < 0.01, ***p < 0.005 (a one-tailed Student’s t-student compared against ST-3).

We performed ChIP, using stressed tomato leaves as the starting tissue and purified anti-ASR1 antibody for the IP assay. The recovered DNA was subjected to high throughput sequencing on Illumina Hiseq 2000 equipment. To identify immuno-enriched regions, we made use of the Macs software program [21] (settings described in Additional file 1: List 1). Macs generated a list of 225 regions enriched in the immunoprecipitated sample; the most statistically relevant are shown in Table 1 (a complete list is given in Additional file 2: Data set 1). To corroborate the informatics analysis, the peaks were manually visualized using the Integrative Genomics Viewer (IGV) genome browser [22] (Figure 2A). Analysis was also performed with the software program Cisgenome [23] and the statistical package CSAR [24], but these programs yielded false-positive peaks (present in both precipitated and INPUT samples) and were thus not used for further analysis. The software program readingExtension was used to extend the reads of the SAM file from approximately 51 to 400 nt (Additional file 3: TomatoProgramCode zip file).

**Table 1:** Peaks derived from the Macs program

**Chromosome**	**Start**	**End**	**Length**	**Score**
1	82955709	82956371	663	306.0
7	1194442	1195252	811	286.97
1	76438827	76439515	689	251.83
5	4911834	4912728	895	236.06
5	4907997	4908831	835	235.12
9	4315702	4316286	585	230.55
1	89187456	89187990	535	214.65
8	61869092	61869619	528	201.54
3	59054967	59055546	580	188.78
9	58915814	58916418	605	184.69
6	41195995	41196596	602	173.86
2	46987902	46988604	703	173.8
2	48205156	48205766	611	173.8
12	6609432	6610083	652	173.8
12	64009107	64009667	561	173.51

The 15 peaks that showed the highest statistical significance (highest scores) as determined by Macs [21] are listed. For each peak, the tomato chromosome number, its boundaries in the sequence of the tomato genome [17], the length and the score are indicated.

### Validation of the immuno-enriched sequences revealed by high throughput sequencing

To validate the accepted peaks resulting from our genome-wide approach, we performed qPCR designed to individually amplify several previously ChIP-enriched regions, chosen due to either the known function of the genes present in the peaks or a high statistical value (Figure 2B, Table 2, Additional file 1: Table S1). An additional peak-free region was used as a negative control. We used a duplicate of the sample subjected to deep sequencing as a DNA template for PCR amplification, in addition to two other independent immunoprecipitates obtained under the same conditions. In all three samples, the five selected amplicons were found to be significantly enriched (using a one-tailed T-student test) when compared to the negative control ST-3 (Figure 2B).

**Table 2:** Amplicons chosen for the validation of ChIP-seq data

**Amplicon**	**Gene**	**Predicted function**
3-830	Solyc03g097830.2	Poly(A) polymerase
9-950	Solyc09g010950.2	Protease in cell division
10-800	Solyc10g054800.1	Aquaporin
10-810	Solyc10g054810.1	Aquaporin
10-820	Solyc10g054820.1	Aquaporin
ST-3	Solyc09g074230.2	Transport of sugars

The names and predicted functions of the ASR1-immuno-enriched genes present in peaks. The non-enriched gene ST-3 was used as a negative control.

### Overall bioinformatics analysis

**Figure 3:**
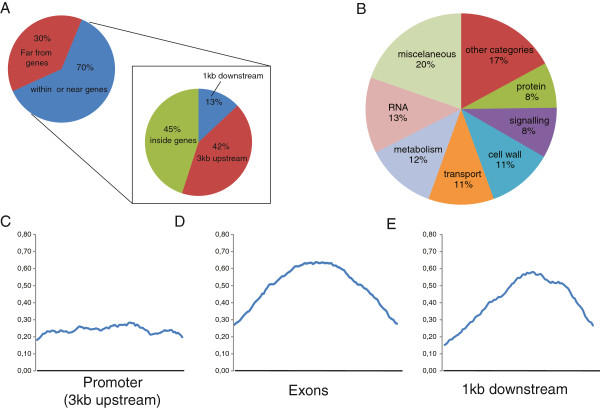
**Analysis of ASR1-bound genomic regions. A)** Distribution of ASR1-binding regions along the genome. The percentage of peaks located 5’ (up to 3 kb upstream of the start point of transcription), within the gene bodies and 3’ (down to 1 kb downstream of the end of transcription). **B)** Cellular functions of the enriched genes present in peaks. Classification was based on reported gene functions. Small categories comprising less than 2% of the genes were grouped and referred to as “other categories” instead of “miscellaneous”. **C-D-E)** Average distribution of leaf ASR1 protein upon stress along distinct tomato gene regions. For each region displayed in A, we considered only those genes that gave reads mainly in each visualized category (66 genes for “promoter” **(C)**, 72 genes for “exons” **(D)** and 21 genes for “downstream regions” **(E)**). Each gene region was subdivided into 100 consecutive segments. The X-axis represents the segment number. The number of reads in each segment (for each gene) was relativized to the number of reads of the gene that showed the highest peak. Normalized values from all the genes for each segment were then averaged (Y-axis) to display any bias in the location of ASR1 binding within each region.

Once we confirmed that our ChIP data were reproducible and confident, we carried out a deeper inspection of the data. We observed that 70% of the 225 regions studied localize to gene-containing regions (the complete list is in Additional file 4: Data set 2). We defined these “genic regions” as spanning from 3 kb upstream of the transcription initiation site to 1 kb downstream of the end of transcription. By further analyzing the peaks found within genes, we observed that 42% of them localized to the upstream regulatory region of the corresponding gene (up to 3 kb upstream of the transcription start point) and 45% of them localized to coding regions plus introns. The small remaining fraction (13%) of peaks fell within 1 kb of the downstream sequences (Figure 3A).

### Average distribution maps of leaf ASR1 along the tomato genome upon stress

To get an overall picture of the distribution of ASR1 binding along different gene zones, we constructed average distribution maps for target genes by using the software program “averageDistribution” (Additional file 3: TomatoProgramCode zip file). While the reads were evenly distributed amongst the promoter regions, the peaks were centered when present in exons or downstream regions (Figure 3C).

### Sorting out ASR1 target genes according to function

Genes associated with ASR1 were classified using the software program Mapman [25]. The sorting was based on possible gene functions, mainly according to sequence homology and the presence of domains of known functions in other organisms. We observed a great diversity of gene groups involved with most of the cell functions (Figure 3B, the complete list is in Additional file 5: Data set 3). We also used Mapman software to define which gene groups were significantly over-represented compared with their relative amount in the whole genome. Over-represented categories included genes encoding cell wall-related proteins and major intrinsic proteins (MIPs), as well as miscellaneous functions (Table 3, Additional file 5: Data set 3).

**Table 3:** Over-represented gene groups according to function

**Function**		**Amount**	**Statistical p**	**% in sample**	**% in genome**	**Over-representation (-fold)**
Cell wall	Cellulose synthase	4	1.38e-05	2.45%	0.08%	31.0
	Beta 1,3-glucano hydrolases	3	5.40e-03	1.84%	0.12%	15.3
	Pectin esterases	3	7.57e-03	1.84%	0.18%	10.2
	Breaking down	4	9.84e-03	2.45%	0.12%	20.2
Transport	Membrane Intrinsic Proteins	5	2.78e-06	3.07%	0.02%	135.7
Miscellaneous		23	1.07e-05	14.11%	0.18%	78.4

The abundance of these groups in our dataset versus their abundance in the tomato genome is shown. Over-representation was calculated as the ratio between the % abundance in immunoprecipitated samples and the % abundance in the whole genome. Statistical p-values are indicated.

### Identification of the consensus ASR1-binding motif

**Figure 4:**
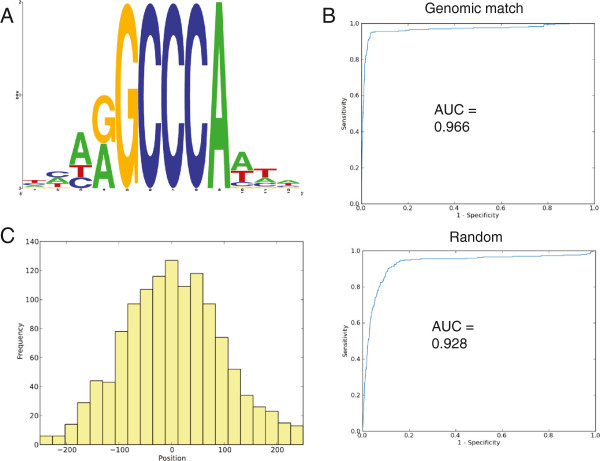
**Consensus ASR1-binding DNA motif.** The consensus sequence was determined by Gimmemotif [26]. **A)** The best consensus sequence motif obtained. The size of each letter is proportional to the frequency of each nucleotide in that position within the consensus motif. **B)** Specificity and sensitivity of consensus sequences tested with a ROC curve using both genomic and random background types. Values of the area under the ROC curve (AUC) near 1 indicate better consensus sequences while values near 0.5 indicate a lack of consensus. **C)** Frequency distribution of hits for the consensus motif relative to the center of the input peaks. The graph indicates that the consensus motif is well centered.

The consensus ASR1-binding motif was determined using the software program Gimmemotif [26] that identifies similar redundant motifs using a “weighted information content” based on similarity scores and clustering using an iterative procedure. Using this computational tool, we found a robust consensus DNA motif for ASR1 (Figure 4A). Gimmemotif yielded several consensus sequences, and we chose the one with the best performance.

To assess performance, Gimmemotif provides a ROC curve [26] that plots the number of true positives (sensitivity) as a function of false positives (specificity) (Figure 4B). The performance is measured as the area below the ROC curve (ABC-ROC), which ranges from 0.5 for the lowest performance to 1 for perfect performance. The ROC curve is obtained by using either a “genomic” background with the same dinucleotide frequency as the input values or a “random” matched background composed of genomic sequences randomly taken from a similar distance to the transcription start site as the input peaks [26]. Finally, the software plotted the location of the obtained consensus motif relative to the center of each peak and showed that the obtained motif is predominantly located in the middle of the reads (Figure 4C).

Additionally, we used the software program "consensusCounter" (Additional file 3: TomatoProgramCode zip file) to determine that the frequency of the consensus motif in all peaks was 1.45 × 10^-2^ (Additional file 6: Data set 4) compared with the 2.64 × 10^-4^ frequency (50-fold difference) expected by chance using the 33.2% GC content in the tomato genome [27].

### The impact of ASR1 binding on target gene expression

**Figure 5:**
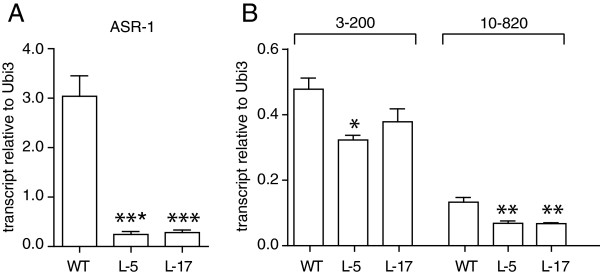
**Stress-driven changes in the expression of identified target genes are ASR1-dependent. A)** Expression levels of Asr1 in WT and two different transgenic ASR1-silenced lines (L-5 and L-17). **B)** Expression levels of 3**–**200 (*Solyc03g115200.2,* Glucan endo-1,3-beta-glucosidase-1) and 10–820 (*Solyc10g054820.1*, Aquaporin) in WT and ASR1-silenced lines. Transcript levels were normalized to the house-keeping gene Ubi3. *p < 0.05, **p < 0.01, ***p < 0.0001 (ANOVA and Bonferroni post-test compared against WT). Two technical replicates and five biological replicates were done.

To determine whether the regulation of the obtained target genes is ASR1-dependent, we compared the expression of two representative target genes in the leaves of Asr1-silenced transgenic plants (two different lines) after a 6-hr water stress treatment (as a 3-hr period was not sufficient to yield significant changes in expression, data not shown). Silenced plants showed a marked decrease (p < 0.0001) in Asr1 mRNA in comparison with WT plants (Figure 5A). When normalizing against the house-keeping gene *Ubi3*, the mRNA levels of gene 10–820 (an aquaporin gene, complete name: *Solyc10g054820.1*) from both transgenic lines were significantly lower (p < 0.01) in comparison with WT plants (Figure 5B). On the other hand, the expression levels of gene 3–200 (a glucan endo-1 3-beta-glucosidase gene, complete name: *Solyc03g115200.2*) were decreased only in one Asr1-silenced line (Figure 5B). Similar results were obtained when mRNA levels were normalized to EF-1, a house-keeping gene (Additional file 1: Figure S3 A and B).

These results, obtained with representatives of cell wall- and aquaporin-associated genes, indicate that the binding of ASR1 to its target genes is truly productive and results in gene regulation upon the induction of water stress, confirming previous hints of ASR1 being a direct transcriptional activator [12,13,28].

The identified consensus motif sequence (Figure 4A) was consistently found three times at the single exon of gene 10–820 and 7 times at the promoter of gene 3–200.

## Discussion and conclusions

For our genome-wide analysis, we carefully followed the current guidelines for ChIP-seq [29] and assessed the quality of the antibody and the robustness of our bioinformatics tools to interpret our high throughput DNA sequencing results. Our ChIP-seq data showed 225 peaks with different values of statistical significance. The results of ChIP-seq were also analyzed with Cisgenome [30] and CSAR [24], but these results gave false peaks and were thus discarded. Artifacts can arise because the annotated sequence of the tomato genome [17] is not error-free, particularly regarding the copy number of repetitive elements and ribosomal genes, which may have been underestimated.

ASR1 showed a binding preference (70%) for gene regions, which is expected for a TF. Considering that tomato genes (including their introns) represent only approximately 13% of the whole genome (even considering non-protein-coding RNA genes) [31], this proportion is even more striking. When we performed a more in-depth analysis of the location of the immuno-enriched sequences that fell into the “gene region” category in the genome, we observed that the majority of them were either upstream of the genes or in the body of the genes, rather than 3’downstream of them. This was not surprising as it is widely accepted that regulatory regions can be found not only at 5’ upstream regions and sizeable distances from the coding region, but also within protein-coding exons as is the case of enhancers of zebra fish developmental genes [32].

A comprehensive analysis of the target genes was made difficult by the diversity of functional categories (at the biochemical or cellular level) encountered. For this reason, we explored over-represented genes whose molecular or cellular functions seemed to be more influenced by ASR1 *a priori*, for example, those encoding cell wall proteins and aquaporins (AQP), which are related to the physiological response to limited water accessibility [33].

Aquaporin genes belong to the Major Intrinsic Protein (MIP) gene family. In plants, the number of AQP genes present in a single species is rather high, with more than 30 AQP genes frequently found in each genome [34]. Plant AQPs are classified into seven different subfamilies according to sequence similarity [35-37]. Studies of AQP expression under drought stress have focused mainly on the PIP (Plasma Membrane Intrinsic Proteins) subfamily and have yielded opposite or conservative results, depending on the isoform analyzed [38-40]. Some reports show that TIPs (tonoplast intrinsic proteins) and XIPs (X intrinsic proteins) subfamilies also modify their expression under abiotic stress [41-43]. The expression of TIPs has been observed in stomatal guard cells and is in agreement with their known role in drought stress [44]. One of the AQP genes we found as target of ASR1 is an ortholog of AtPIP1;4 (NP_567178.1), frequently expressed in leaves and flowers. Consistent with our results, this *Arabidopsis* gene has been reported to be up-regulated during drought stress [39]. Moreover, transcript levels of the tomato aquaporin gene *Solyc10g054820.1* were found lower in ASR1-silenced plants compared to WT ones, thus validating the results of ASR1 binding at a functional level. In agreement with an expected delay between TF binding and effective transcription due to chromatin remodeling [45], such differences in expression were not detected at the 3 hours of stress chosen for the ChIP experiments but after 6 hours instead.

The plant cell wall provides structural support during development and represents the first line of defense against biotic and abiotic stressors, including drought. In recent years, evidence has accumulated for a dedicated maintenance mechanism for plant cell integrity under diverse biotic and abiotic stress; however, the underlying mechanism remains to be elucidated. Thus, the discovery of cell wall-related genes as targets of ASR1 makes sense in light of the complex network of polymers essential for maintaining turgor pressure. Water loss in this matrix results in a severe disruption of cell wall integrity, which can be irreversible [33]. In addition, because turgor pressure is fundamental to plant cell growth [46], it is conceivable that tissues in active growth (i.e., apical parts) make their walls more extensible, while other tissues (i.e., cell walls) harden, allowing for continuous growth under low water potentials [47]. It is interesting that the “cell wall” gene group we identified includes genes involved in cellulose synthesis, cell wall breakdown and remodeling.

In particular, we found that ASR1 binds directly to several cell wall-related genes. One such gene is *Solyc08g082650.2.1*, which is annotated as a cellulose synthase-like (CSL) protein. The three closest genes related to Solyc08g082650.2.1 in *Arabidopsis thaliana* are the paralogous cellulose synthase-like G (CSLG) proteins 2 (AtCSLG2, At4g24000) and 3 (AtCSLG3, At4g23990), which contain a relatively high degree of identity (48-50%) in the region between aa 26–205 (p range = 5 - 6e-43). By searching the GENEVESTIGATOR platform (http://www.genevestigator.com), we found that AtCSLG2/G3 appear to be active during leaf senescence and that osmotic or water stress stimulates their expression up to 50-fold.

Another cell wall-associated gene that emerged as a direct target of ASR1 is Solyc03g115200.2.1, which is annotated as Glucan endo-1,3-β-glucosidase 1. The closest ortholog in *Arabidopsis thaliana* is the *plasmodesmata callose-binding protein 3* (AtPDCB3) gene, with a high degree of identity (52%; p = 1.0e-43). AtPDCB3 exhibits specific callose-binding activity *in vitro* and localizes to plasmodesmata [48]. Deposition of callose (a β-1,3-glucan) at plasmodesmata is known to be stimulated by physical and physiological stresses [49,50]. Interestingly, the GENEVESTIGATOR database reveals that the expression of AtPDCB3 is highest in seeds during desiccation and stratification, reinforcing the concept of a conserved response of this kind of proteins to restricted water availability in both *Arabidopsis* and tomato.

Another work seeking targets of tomato ASR1 was able to identify ABI4, a gene involved in seed germination [14]. However, this study suffers from the fact that it has been conducted in transgenic Arabidopsis, with a smaller genome, thus missing many possible target genes.

Our results also revealed a consensus *in vivo* ASR-binding DNA motif with little variation: (A/T)(A/G)GCCCA, almost identical to the one very recently found for ASR5 in rice subjected to Aluminium stress [51] and to the one described for the TF AtTCP20 [52] (Table 4), whose targets are cytochrome genes in *Arabidopsis*[53]. It is thus tempting to speculate that AtTCP20 may function as the counterpart to ASR1 in *Arabidopsis*, which would be consistent with the known connection between adaptation to abiotic stress and oxidative respiration through mitochondrial electron transport in plants [54]. Interestingly, we also identified four target paralogous genes of ASR1 encoding Cytochrome P450. A related Cytochrome P450 functioning as an ABA-8’-hydroxylase is known to inactivate ABA [55], a paradigmatic plant hormone involved in the response to drought stress. Because ABA induces ASR1 expression and the mentioned enzyme catalyzes the first step in the oxidative degradation of ABA, ASR1 might regulate ABA endogenous levels in a feedback fashion under water stress situations.

**Table 4:** Other transcription factors with a similar DNA-binding motif

**Database**	**TF**	**E value**	**Alignment**
PLACE	AtTCP20 (SITEIIATCYTC)	8.4270e-10	WRGCCCA
			-RGCCCA
	minus284MOTIFZMSBE1	2.0664e-06	-----TGGGCYW-----
			TCTGGGCCGATTGGCCTTTGGGCTTGCA
	GCBP2ZMGAPC4	8.1033e-06	-WRGCCCA
			CGGGCCCAC
	SITEIIAOSPCNA	8.1033e-06	--WRGCCCA
			ACGGGCCCA
	UP1ATMSD	8.1033e-06	---TGGGCYW
			WWWTGGGCC
TRANSFAC FAMRS	bHLH_PCF2_M00948	1.5189e-05	-TGGGCYW
			GTGGGNCCN
	CC_HNF4,_M00967	3.9030e-05	-TGGGCYW
			NTGGACYT
	homeo_Pitx2_M00482	1.3290e-04	-WRGCCCA
			NTAATCCCAN
	CH_Egr-3_M00245	3.9782e-04	WRGCCCA----
			ACGCCCACGCA
	CH_Egr-1_M00243	6.9656e-04	WRGCCCA----
			MCGCCCACGCA
AGRIS	SORLIP2	1.5219e-05	WRGCCCA
			-GGCCC
ATHAMAP	PCF2	3.0241e-05	-TGGGCYW--
			GTGGGNCCNN

The ASR1-binding DNA sequence was compared to four different databases using the STAMP program [56]. Columns show the databases used for comparison, the TFs possessing similar binding sites, the statistical E values and the alignments between the ASR1 consensus sequence found in this study (on top) as well as for the other TFs (bottom) for each hit, respectively. Single letter codes for more than one nucleotide at ambiguous positions are the same as recommended in http://www.ncbi.nlm.nih.gov/staff/tao/tools/tool_lettercode.html. All of the computer programs compared both plus and minus strands. ASR1 showed a binding sequence almost identical to the one described for SITEIIATCYTC (AtTCP20).

In addition, the motif we found shows partial coincidence with the one obtained by SELEX [57]. It is noteworthy that while ChIP captures sequences *in vivo*, SELEX is carried out *in vitro* with ADN devoid of histones, an artificial condition. On the other hand, our ChIP data shows no enrichment of promoters/enhancers of genes orthologous to grape VvHT1, a sugar transporter gene reported to be a target of ASR [58]. This result is consistent with the absence of the consensus motif in VvHT1 and its presence—repeatedly up to eight times—in genomic regions highly enriched by ChIP. Here, it is important to note that [13] used a heterologous system (yeast) to test a plant protein-DNA interaction and that neither the grape ASR protein nor the VvHT1 gene are identical to their counterparts in tomato.

At this point, it is worth mentioning the scope of ChIP in general. This procedure, due to its crosslinking step, is also able to detect proteins indirectly binding to DNA, for example by forming a DNA-interacting complex. Nevertheless, even in this second scenario, the information gained by ChIP is useful. The way to discriminate between direct and indirect binding is to perform *in vit*ro assays with the purified TF, in this case ASR1, at the expense of losing the *in vivo* context (native chromatin), which has already been performed [14,57].

## Conclusions

In summary, we have uncovered a novel repertoire of target genes of the TF ASR1, some of which are clearly involved in the response and physiological adaptation of plants to water stress. These findings will hopefully enable us to gain additional insight into both the early environmental stress-sensing molecular events triggered by ABA and the late physiological adjustments that finally confer tolerance to drought.

## Methods

### WT plants

Commercial tomato (*Solanum lycopersicum*) seeds were germinated on blotting paper for 7 days and then transferred to pots containing soil mix in a growth chamber under a photoperiod of 16 hr light/8 hr darkness at 26°C. Plants were used 4 weeks later.

### Stress conditions

Soil was carefully removed from the roots with the aid of a small amount of water to minimize damage. Plants were then stressed by being placed onto blotting paper under an incandescent lamp for 3 hr. Some plants were re-watered to confirm reversibility of stress and healthy recovery. Young leaves were then cut and immediately frozen in liquid nitrogen for subsequent purification of nuclei as the starting material for ChIP (see below). For expression analysis, the procedure was the same except that we applied a 6-hr water stress treatment, as a 3-hr period was insufficient to yield significant changes in expression.

### Asr1-silenced transgenic plants

Tomato (*Solanum lycopersicum*) L. cv Moneymaker seeds were obtained from Meyer Beck (Berlin), and the plants were handled as previously described [59]. The 348-bp coding region of the tomato Asr1 gene (GenBank U86130.1) was cloned in antisense orientation into the multiple cloning site of the pBINAR vector [60] between the Cauliflower mosaic virus 35S promoter and the octopine synthase terminator. The construct was delivered by *Agrobacterium tumefaciens* into tomato cotyledons. Emerging shoots were excised and selected on Murashige and Skoog media containing kanamycin (100 mg/l). When the plants developed roots, they were transferred to soil in the greenhouse for subsequent selection. The initial screening for the 39 lines was based on a diminished expression displayed in Northern blots. For the expression assays, we used five WT tomato plants and 10 Asr1-silenced plants (five from line 5 and five from line 17).

### ChIP-sequencing (ChIP-seq)

This strategy combines chromatin immunoprecipitation (ChIP) with high throughput DNA sequencing to identify the binding sites of nuclear proteins connected non-covalently to DNA. A key success factor is the generation of a high-quality antibody against the purified protein of interest. In this work, ChIP-seq was chosen to identify the targets of ASR1 starting out with the purification of ASR1, against which antibodies were produced.

### Expression of recombinant ASR1 in *Escherichia coli* BL21

Expression of recombinant ASR1 was achieved using a plasmid (PRSET B vector, Invitrogen) that contains the tomato Asr1 cDNA driven by the prokaryote promoter sequence recognized by the RNA polymerase of bacteriophage T7 (construct T7-ASR1). The recombinant plasmid was introduced into the *Escherichia coli* BL21 strain, which has a lac-driven T7 RNA polymerase gene. For testing induction, low-scale cultures were started and Asr1 was indirectly induced by IPTG at different times. As a negative control, a culture of plasmid-free *E. coli* BL21 was used. Crude protein extracts were run in 15% PAGE, transferred to a nitrocellulose membrane and stained with Coomassie Blue.

### Purification of ASR1 by affinity chromatography

Cultures induced by IPTG for 2 hr were lysed through sonication, and recombinant ASR1 protein was purified with a pre-packed Ni^2+^column (HisTrap Kit, Pharmacia Biotech) using its natural histidine-rich tract as a tag. The ASR1 protein appeared to be pure, with no detectable contaminant proteins, after elution through a non-linear continuous gradient of imidazol (Additional file 1: Figure S1).

### Antibody raised against pure ASR1

Purified ASR1 was concentrated through 10-kDa cutoff spin columns (Vivaspin, GE Healthcare) and inoculated into rabbits. Bleedings were performed for titrations of antisera.

### Purification of total immunoglobulins from rabbit antisera

Total immunoglobulins from antisera were precipitated with ammonium sulfate, dialyzed and purified with a “Hi-trap protein G” column (Pharmacia Biotech). Their presence was monitored through absorbance at 280 nm. Immunoglobulin-rich fractions were collected and concentrated by Vivaspin columns (GE Healthcare).

### Purification and checking of the anti-ASR1 antibody

The purified antibodies were diluted in PBS to conduct an affinity chromatography protocol using ASR1 covalently bound to a cyanogen-activated Sepharose solid matrix (C9210 beads, Sigma). Dot blots and subsequent Ponceau Red staining were performed to visualize the eluted fractions that contained the protein material (Additional file 1: Figure 2)—the pure and specific antibody that was later tested by immunoprecipitation (see two paragraphs below).

### ChIP protocol

The ChIP protocol was performed as described in [61] from nuclei of water stressed tomato leaves. For DNA fragmentation, we used a Biorruptor UCD-200 TM machine (Diagenode, Denville, NJ, USA) (30 cycles at max power, 30 sec ON/30 sec OFF).

### Immunoprecipitation and Western blot

To test the quality (the specificity and precipitation capacity towards ASR1) of the antibody, we first performed a non-crosslinking ChIP procedure using fruit as the starting tissue. The protocol was stopped at the crosslinking reversal step. Precipitated proteins were eluted with 0.1 M glycine at a pH of 2.4. The sample was then transferred into a tube containing enough 1 M Tris-base to neutralize the low pH and loaded onto an SDS-PAGE gel for subsequent Western blot to detect the immunoprecipitated ASR1. Because the same primary anti-ASR1 antibody was used for both IP and Western blot, for the latter we used a secondary anti-native rabbit immunoglobulin TrueBlot antibody (Rockland, USA) to avoid the detection of immunoglobulins that had been run (denatured) on the gel and whose quality was tested in this assay.

### Construction of the DNA fragment library for deep sequencing

Following post-stress leaf chromatin immunoprecipitation, the DNA was purified using “AMPXP” beads according to the manufacturer’s protocol (Ambion). The DNA was eluted in 10 μl MilliQ water followed by two repeat purifications. The DNA fragments were refilled to get blunt ends using the Klenow fragment of DNA polymerase. Fragmented ends were phosphorylated by T4 kinase and ligated to different double-stranded adaptors by concentrated DNA ligase. The adaptors had 5 nucleotides in their 3′ ends as “bar codes” for sample identification in multiplex runs. The DNA was re-purified with shorter incubation times to favor the loss of adaptor primer dimers shorter than 200 bp that were generated during ligation. PCR reactions were performed using 0.5 μl of dimer-free adaptor samples, adaptor-specific primers and high-fidelity thermostable DNA polymerase (Phusion Hot Start II, Thermo Scientific) with low cycle numbers (between 12 and 18) until a product concentration of 5 ng/μl was reached. Cycling conditions were as follows: 1 starting denaturation cycle of 30 sec at 98°C, 12–18 cycles of 10 sec at 98°C (denaturing), 30 sec at 65°C (annealing), 30 sec at 72°C (elongation) and a final elongation step for 5 min at 72°C. Amplified products with each bar code were combined and re-purified with beads and resuspended. The concentration of each sample for sequencing was 2 ng/μl.

### Deep sequencing proper

DNA samples from the library were sequenced on a HiSeq Illumina® machine (Illumina, San Diego, CA, USA) at BTI, Cornell University campus, Ithaca, NY, USA).

### Data access

The raw sequencing data have been uploaded at the following NCBI website, publicly available: http://www.ncbi.nlm.nih.gov/sra/?term=SRX257002.

### Bioinformatics tools

The reads were processed into separate sequences according to bar codes and trimmed by using the fastx toolbox software (http://cancan.cshl.edu/labmembers/gordon/fastx_toolkit/). Sequences were aligned with those from the tomato genome (version SL2.40) available on the webpage of the Genome Project [17] (http://solgenomics.net/organism/Solanum_lycopersicum/genome) using the software program Bowtie [62]. Reads that produced two mismatches when aligned were discarded, as were reads that aligned with more than one position in the genome. The reads were sorted and indexed with the aid of SAMtools (Sequence Alignment Map format) at the Boyce Thompson Institute for Plant Research. Finally, the number of sequences that fell within each 150-bp window was counted and visualized with IGV [22]. For peak calling, data were analyzed using the Macs program [63] (parameters listed in Additional file 1: List 1), which searches for peaks (regions that yielded a high number of reads) present in the sample but not in the control (input sample). We also used the CSAR [24] and cisgenome [23] programs, which use the statistical programming language R [64]. The consensus motif was determined by the Gimmemotif program [26]. Over-represented gene categories were resolved with the Mapman program [25] (parameters listed in Additional file 1: List 1). The ASR1-binding sequence was compared to four different databases using the STAMP program [56].

The in-house software programs “averageDistribution”, “consensusCounter” and “readingExtension” were created to make our software freely available. The source code was uploaded as Additional file 3: (in the zip folder “TomatoProgramCode”). For each program, there is a folder including a readme.txt file containing a brief explanation about what it does and how to execute the program, as well as a description of the contents of each file.

We used the STAMP program [56] to search databases for additional TFs with similar DNA-binding motifs as the one recognized by ASR1.

### Individual real time PCRs for the validation of deep-sequencing results

The reactions were carried out with purified immunoprecipitated DNA and Recombinant Taq DNA polymerase (Invitrogen) in a final volume of 25 μl. A DNA Engine Opticon (MJ Research Inc.) thermocycler was used with annealing temperatures set to achieve 90% amplification efficiency (between 59°C and 64°C, depending on the primers). Denaturation curves were calculated and the amplified DNA was run through an agarose gel to ensure the existence of a single product. The conditions were as follows: 1 cycle of 5 min at 94°C (initial denaturation), 35 cycles of 30 sec at 94°C (denaturation), 30 sec at 59/64°C (annealing) and 30 sec at 72°C (elongation).

### Expression analysis of target genes

For RNA extraction, we used the TriReagent kit (MRC Inc.) with 300 mg of previously mortar-ground leaves as a starting material and 1.5 ml of TriReagent solution according to the manufacturer’s protocol. All RNA samples were quantified using a Nanodrop 2000 (Thermo Scientific) spectrophotometer. To eliminate contamination of the samples by residual DNA, 10 μg of each RNA sample were treated with 12.5 U DNAseI (Invitrogen). Reverse transcription was achieved using 2 μl of DNAseI-treated RNA, 50 U MMLV-RT (Promega) and oligo-dT (50 pmoles) in a 25 μl final volume for 1 hr at 42°C. To prevent RNA degradation, 10 U of RNAseOUT (Invitrogen) was added. Following reverse transcription, qPCR was carried out using 5 μl of a 1/10 dilution of the cDNA samples (obtained as described above) per PCR reaction. Reactions were performed in a DNA Engine Opticon (MJ Research Inc.) thermocycler. We used 0.625 U of Taq DNA Polymerase (Invitrogen), 3 mM magnesium chloride, 2 mM of dNTPs mixture (Fermentas) and 0.2 μM of each primer (IDT Inc.) in a final volume of 25 μl. We used Sybr Green® (Roche) as the fluorophore. Reactions were conducted under the following cycling conditions: 2 min of denaturation at 94°C, 40 cycles of 30 sec of denaturation at 94°C, 30 sec of annealing and 30 sec of elongation at 72°C. A melting curve was generated between 65 and 95°C with readings at every 0.5°C. For Asr1, Ubi3 and EF-1 quantitation we used 67°C as the annealing temperature. For the *Solyc03g115200.2* gene (3–200), we used 58.6°C and for the *Solyc10g054820.1* gene (10–820), we used 62.4°C. qRT-PCR was validated by a standard and melting curve. Primers are listed in Additional file 1: Table S1. For each amplicon, a standard curve was made. Quantitation of DNA in each sample was extrapolated from its respective standard curve. Values were then relativized to that of a housekeeping gene calculated in the same way [65]. Levels from Asr1, *Solyc03g115200.2* (3–200) and *Solyc10g054820.1* (10–820) were normalized to *Ubi3* or *EF-1* levels. Statistical analysis was performed with the GraphPad software program using a one-way ANOVA statistical test with a 95% confidence level.

### Description of additional data files

The Data set 1 contains a table with the list of peaks found with the Macs software. Data set 2 shows a list of all the genes with peaks near them. Data set 3 shows a list of the gene categories, the over-represented functions and the over-represented groups obtained after running Mapman software. Data set 4 shows the count of reads for ASR1-binding sequences along all the peaks found by the Macs software. The Additional file 1: (pdf) contains 3 figures, 2 tables and 1 parameter list. Figures 1 and 2 show the results of ASR1 protein purification and anti-ASR1 antibody affinity chromatography. Figure 3 shows qRT-PCR experiments using a second housekeeping gene to express the relative amounts of mRNAs. Tables 1 and 2 list primers used for ChIP-qPCR and qRT-PCR, respectively. Finally, the Additional file 1: List 1 shows the parameters used for Macs and Gimmemotif softwares.

The scripts and in-house softwares developed for this paper are included in the Additional file 3: “tomatoProgramCode.zip” separate file.

## Abbreviations

MYA: Million years ago; LEA: Late embryogenesis abundant; ABA: Abscisic acid; ASR: ABA/ Stress/Ripening (lower case: gene; upper case: protein); ChIP: Chromatin immunoprecipitation; DNA: Deoxyribonucleic acid; cDNA: Complementary DNA; TF: Transcription factor; ROC: Receiver operating characteristic; MIP: Major intrinsic proteins; AQP: Aquaporins; WT: Wild type.

## Competing interests

All the authors declare that they have no competing interests.

## Authors’ contributions

MMR and RMG (equal contributions) performed all the experimental work on Asr1 purification, antibody making, ChIP for deep sequencing as well as subsequent ChIP-qPCR and qRT-PCR for validation of individual loci and transcripts; JJG and SZ provided the equipment and setups for library making and deep sequencing (SZ made the libraries); TD carried out the initial bioinformatic analyses; PGT developed in-house softwares for in-depth bioinformatic interpretation of the data; FC and PGD generated the ASR1-silenced plants used for functional validation; KA interpreted the results regarding aquaporin genes; JME interpreted the results regarding cell wall-related genes, JDSS made valuable suggestions throughout the work; NDI introduced the theoretical frame, coordinated the project and drafted the manuscript. All authors read and approved the final manuscript.

## Supplementary Material

Additional file 1: Figure S1 Purification of ASR1 by a Ni^2+^ affinity column chromatography. **Figure S2.** Purification of the anti-ASR1 antiboby. **Figure S3.** Testing another housekeeping gene for normalization of transcript levels. **Table S1.** Primers used for ChlP validation. **Table S2.** Primers used for expressions analysis (qRT-PCR). **List 1.** Software parameters used in this work. Click here for file

Additional file 2 **Data set 1.** Table with the list of peaks found with the Macs software. Click here for file

Additional file 3 **TomatoProgramCode zip file.** In-house softwares developed for this paper. Click here for file

Additional file 4 **Data set 2.** List of all the genes with peaks in or near them. Click here for file

Additional file 5 **Data Set 3.** List of the gene categories, the over-represented functions and the over-represented groups obtained after running the Mapman software. Click here for file

Additional file 6 **Data set 4.** Count of reads for ASR1-binding sequences along all the peaks found by the Macs software. Click here for file
