# Supplementary material for: Genome-wide data (ChIP-seq) enabled identification of cell wall-related and aquaporin genes as targets of tomato ASR1, a drought stress-responsive transcription factor
Source: BMC Plant Biol. 2014 Jan 14;14:29. doi: 10.1186/1471-2229-14-29 (PMC3923394; doi:10.1186/1471-2229-14-29)
Supplement: Additional file 1: Figure S1 — Purification of ASR1 by a Ni2+ affinity column chromatography. Figure S2. Purification of the anti-ASR1 antiboby. Figure S3. Testing another housekeeping gene for normalization of transcript levels. Table S1. Primers used for ChlP validation. Table S2. Primers used for expressions analysis (qRT-PCR). List 1. Software parameters used in this work. [file 1471-2229-14-29-S1.pdf]

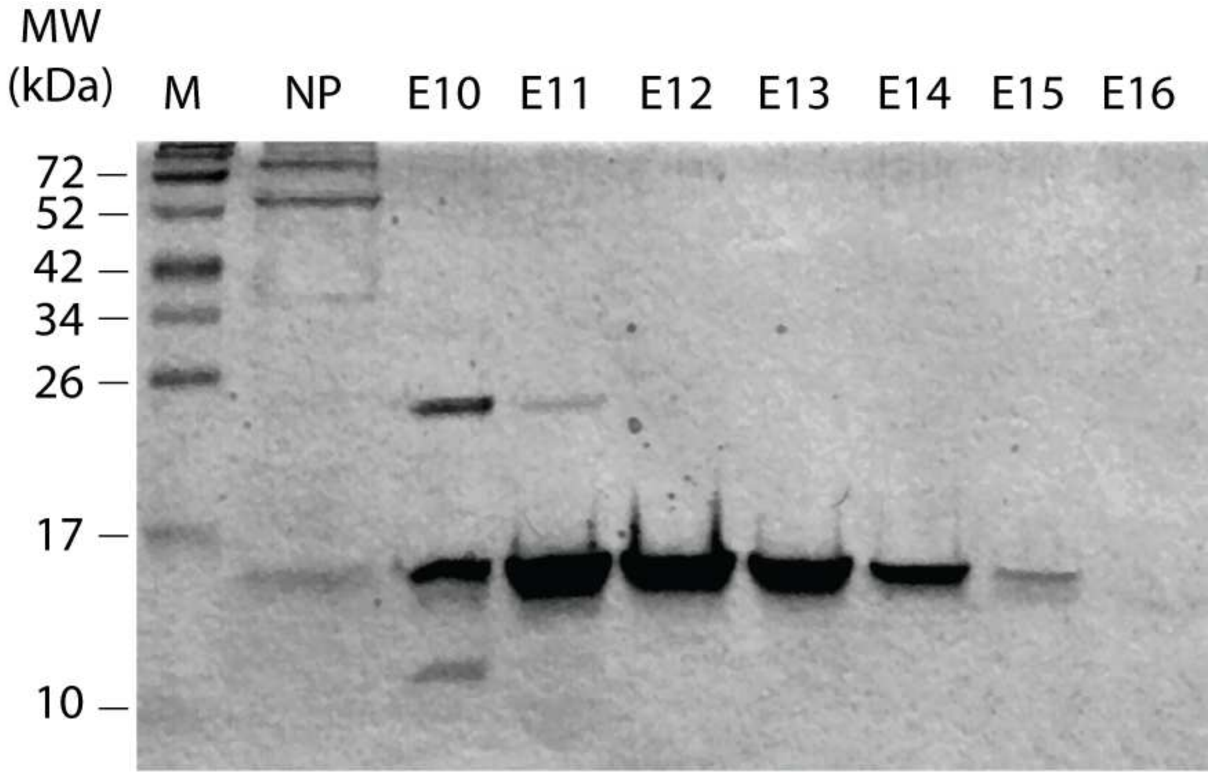

Suppl. Fig. 1. Purification of ASR1 by a Ni^{2+} affinity column chromatography. SDS-PAGE of fractions eluted off the column through a non-linear continuous gradient of imidazol. ASR1 appeared pure in fractions E12-E15, with no detectable contaminant proteins. MW: Molecular weight markers. NP: non-purified starting material from recombinant *E. coli* cultures.

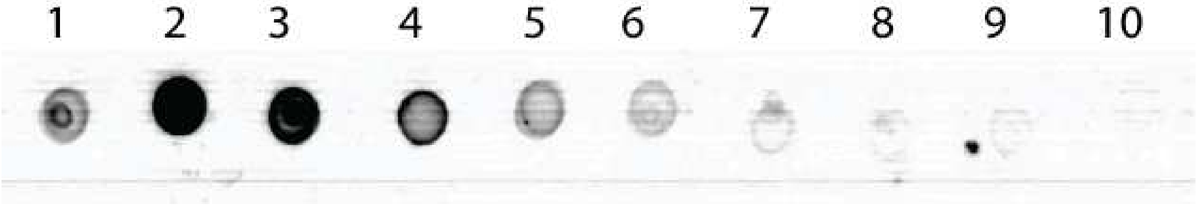

Suppl. Fig. 2. Purification of the anti-ASR1 antibody. Total immunoglobulins from ASR1-immunized rabbits were precipitated with ammonium sulphate, dialyzed and passed through a Hi-Trap Protein G column. Eluted immunoglobulin fractions showing high O.D. measurements at 280 were then pooled and passed through a prepared Sepharose-ASR1 column. 1 ul of the successive eluted fractions was spotted on a nitrocellulose strip. After drying, Ponceau Red staining was performed to visualize the fractions that revealed a high yield of protein (dot blots 2-4 on the figure representing fractions 2-4).

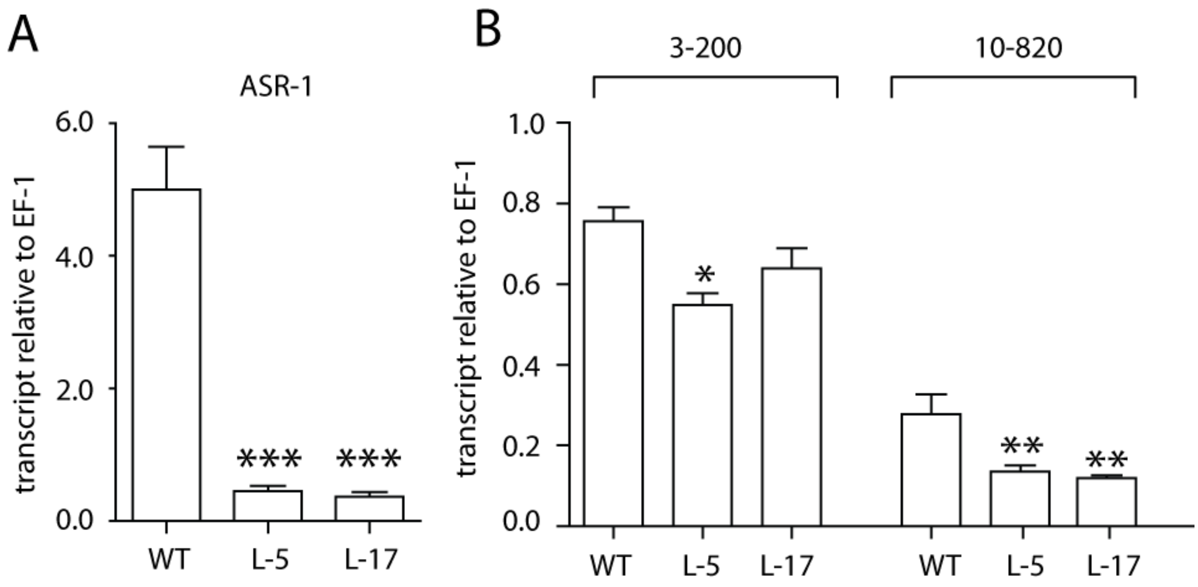

Suppl. Fig 3. Testing another housekeeping gene for normalization of transcript levels. Expression levels of Asr1 (A), *Solyc03g115200.1* (Glucan endo-1 3-beta-glucosidase and *Solyc10g054820.1* (aquaporin) (B) genes, relativized against the house-keeping gene EF-1 in WT and Asr1-silenced (L-5 and L-17 lines) plants. *p<0.05, **p<0.01, ***p<0.0001.

Amplicon	Primers	Sequence	PCR Efficiency
Solyc09g010950.2 (9-950)	Fw	TAACCCTTGTGAGCCCATTC	91.10%
	Rev	AGTTGATGAAAGCCCAGCAC	
Solyc10g054800.1 (10-800)	Fw	TTGCCCTATGGCAGAAAGAG	89.50%
	Rev	TAGCCCGGACTCAATAATGG	
Solyc10g054810.1 (10-810)	Fw	GCCTCTTTTAGCGGTGATTG	97.50%
	Rev	TTGGCCTTGTCACTGTCTTG	
Solyc10g054820.1 (10-820)	Fw	AAGGAAACCCCATACATCACC	99.30%
	Rev	GGGTTGTTAAGGCTTTGTCG	
Solyc03g097830.2 (3-830)	Fw	TGGACCGATGACATCTTAGC	98.80%
	Rev	AAGACTCTGGACTCGGGTTG	
Solyc05g008080.1 (5-080)	Fw	TCCTCGAACATGACTCGAAC	90.50%
	Rev	TGGCCCAATACTTCTTGCTG	
Solyc05g056130.2 (5-130)	Fw	AACAAGCTTTGGGCTTTGG	93.00%
	Rev	TGTTGGCCCAGTCTATTGAG	
Solyc07g062660.2 (7-660)	Fw	CCCAAAGCAACAGTTGTCAC	94.00%
	Rev	GGGCTTCTAGGCCTGTATTTTC	
ST-3	Fw	GGATGAGCATCATATGCGTAG	98.80%
	Rev	TTTCACACGTCTCTGCCAAG	

Suppl. Table 1. Primers used for ChIP validation.

Gene	Primers	Sequence	PCR efficiency
Asr1	Fw	CAGATGGAGGAGGAGAAACAC	90.20 %
	Rev	TAGAAGAGATGGTGGTGTCCC	
Solyc03g115200.2 (3-200)	Fw	CTTCATACAAACGGGCCTTG	97.00 %
	Rev	GTTGGTTGTGGTTGTGGATG	
Solyc10g054820.1 (10-820)	Fw	AAGGAAACCCCATACATCACC	96.40 %
	Rev	GGGTTGTTAAGGCTTTGTCG	
Ubi3	Fw	GCCGACTACAACATCCAGAAGG	97.70 %
	Rev	TGCAACACAGCGAGCTTAACC	
EF-1	Fw	GATTGGTGGTATTGGAACTGTC	99.70 %
	Rev	AGCTTCGTGGTGCATCTC	

Suppl. Table 2. Primers used for expression analysis (qRT-PCR).

Macs Parameters
This file is generated by MACS version 1.4.2 20120305
ARGUMENTS LIST:
name = m_chip
format = AUTO
ChIP-seq file = chip_a8.bam
control file = input.bam
effective genome size = 7.00e+08
band width = 200
model fold = 10,30
p-value cutoff = 1.00e-05
Large dataset will be scaled towards smaller dataset.
Range for calculating regional lambda is: 1000 bps and 10000 bps
tag size is determined as 51 bps
total tags in treatment: 6977570
tags after filtering in treatment: 6977570
maximum duplicate tags at the same position in treatment = 1
Redundant rate in treatment: 0.00
total tags in control: 2071128
tags after filtering in control: 2071128
maximum duplicate tags at the same position in control = 1
Redundant rate in control: 0.00
d = 54

Gimmemotif parameters
Lwidth: 500
Background: genomic_matched, random
Tools: MDmodule, MEME, MotifSampler, trawler, Improbizer, BioProspector, Weeder
weird_option: False
use_strand: False
abs_max: 1000
analysis: large
enrichment: 1.5
width: 200
keep_intermediate: False
genome: Slyco
fraction: 0.2
cluster_threshold: 0.95
max_time: None
available_tools: MDmodule, MEME, GADEM, MotifSampler, trawler, Improbizer, BioProspector, Weeder
user_background: None
p-value: 0.05
markov_model:

Suppl. List 1. Software parameters used in this work.
